# Endotracheal intubation in trauma patients with isolated shock: universally recommended but rarely performed

**DOI:** 10.1007/s00068-022-01988-x

**Published:** 2022-05-12

**Authors:** Timo Stausberg, Tobias Ahnert, Ben Thouet, Rolf Lefering, Andreas Böhmer, Thomas Brockamp, Arasch Wafaisade, Matthias Fröhlich, the TraumaRegister DGU

**Affiliations:** 1 Department of Trauma and Orthopedic Surgery, Cologne-Merheim Medical Centre (CMMC), University of Witten/Herdecke, Ostmerheimerstr.200, 51109 Cologne, Germany; 2 Institute for Research in Operative Medicine (IFOM), University of Witten/Herdecke, Cologne, Germany; 3 Department of Anaesthesiology and Intensive Care Medicine, Cologne-Merheim Medical Centre (CMMC), University of Witten/Herdecke, Cologne, Germany; 4 Committee on Emergency Medicine, Intensive Care and Trauma Management (Sektion NIS) of the German Trauma Society, Berlin, Germany

**Keywords:** Hemorrhagic shock, Airway management, Multiple trauma, Preclinical treatment

## Abstract

**Purpose:**

The indication for pre-hospital endotracheal intubation (ETI) must be well considered as it is associated with several risks and complications. The current guidelines recommend, among other things, ETI in case of shock (systolic blood pressure < 90 mmHg). This study aims to investigate whether isolated hypotension without loss of consciousness is a useful criterion for ETI.

**Methods:**

The data of 37,369 patients taken from the TraumaRegister DGU® were evaluated in a retrospective study with regard to pre-hospital ETI and the underlying indications. Inclusion criteria were the presence of any relevant injuries (Abbreviated Injury Scale [AIS] ≥ 3) and complete pre-hospital management information.

**Results:**

In our cohort, 29.6% of the patients were intubated. The rate of pre-hospital ETI increased with the number of indications. If only one criterion according to current guidelines was present, ETI was often omitted. In 582 patients with shock as the only indication for pre-hospital ETI, only 114 patients (19.6%) were intubated. Comparing these subgroups, the intervention was associated with longer time on scene (25.3 min vs. 41.6 min; *p* < 0.001), higher rate of coagulopathy (31.8% vs. 17.2%), an increased mortality (8.2% vs. 11.5%) and higher standard mortality ratio (1.17 vs. 1.35). If another intubation criterion was present in addition to shock, intubation was performed more frequently.

**Conclusion:**

Decision making for pre-hospital intubation in trauma patients is challenging in front of a variety of factors. Despite the presence of a guideline recommendation, ETI is not always executed. Patients presenting with shock as remaining indication and subsequent intubation showed a decreased outcome. Thus, isolated shock does not appear to be an appropriate indication for pre-hospital ETI, but clearly remains an important surrogate of trauma severity and the need for trauma team activation.

## Introduction

Endotracheal intubation (ETI) is a commonly performed intervention in the pre-hospital treatment of severely injured trauma patients. In Germany, approximately 20% of all severely injured trauma patients got ETI by emergency medical physician [1]. Nevertheless, pre-hospital ETI is associated with several risks and complications, including hypoxemia, injury of the respiratory tract, hypotension and cardiovascular arrest due to anesthesia [2, 3]. The procedure not only results in a longer time on scene but can also have negative impact on the clinical course with a higher rate of multiple organ failure and a longer stay on the intensive care unit [4].

The German trauma guidelines [5] recommend that pre-hospital ETI should be performed in trauma patients with the following indications:

- Bradypnea (respiratory rate < 6/min).

- Hypoxia (SpO_2_ < 90%) despite oxygen therapy and after exclusion of a tension pneumothorax.

- Severe brain injury (GCS < 9).

- Trauma-associated persistent hemodynamic instability (systolic blood pressure (SBP) < 90 mmHg, age-adapted in children).

- Severe chest trauma with respiratory insufficiency (respiratory rate > 29/min, age-adapted in children).

These recommendations are largely in line with the Eastern Association for the Surgery of Trauma practice management guideline and the Scandinavian SSAI clinical practice guideline on pre-hospital airway management [6, 7]. At least, they all have in common that an ETI is indicated in the presence of hemodynamic instability.

Tension pneumothorax, cardiac tamponade and spinal shock can cause hypotension following trauma, but it is mainly caused by blood loss and consecutive hemorrhagic shock.

With exception of spinal shock, hypotension following trauma is usually caused by blood loss and consecutive hemorrhagic shock. In this case, a load-and-go tactic should generally be aimed for and pre-hospital interventions should be limited to the lowest possible level. Necessary steps are to identify and, if possible, control bleeding (for example by compression or tourniquet), avoid hypothermia in favor of the coagulation system and immediate transport to an adequate trauma center for definitive care and surgical bleeding control [8]. Basic measures such as oxygenation and establishment of at best two large-bore intravenous lines are recommended [9].

Pre-hospital interventions such as ETI or chest tubing do not influence the total trauma resuscitation time, which is defined as time until completion of care in the emergency room [10]. However, a prolonged pre-hospital resuscitation time is associated with increased all-cause mortality [11]. Even though emergency medical service considered ETI necessary but failed in securing one, there were still good survival rates [12].

Aim of the study was to analyze whether patients benefit from an ETI due to severe shock. It is hypothesized, that the ETI does not influence the patients’ outcome as long as no other indication for an advanced airway is given.

## Methods

The TraumaRegister DGU® of the German Trauma Society (Deutsche Gesellschaft für Unfallchirurgie, DGU) was founded in 1993. The aim of this multi-center database is a pseudonymized and standardized documentation of severely injured patients.

Data are collected prospectively in four consecutive time phases from the site of the accident until discharge from hospital: (A) pre-hospital phase, (B) emergency room and initial surgery, (C) intensive care unit and (D) discharge. The documentation includes detailed information on demographics, injury pattern, comorbidities, pre- and in-hospital management, course on intensive care unit, relevant laboratory findings including data on transfusion and outcome of each individual. The inclusion criterion is admission to hospital via emergency room with subsequent ICU/ICM care or admission with vital signs and death before admission to ICU. The infrastructure for documentation, data management, and data analysis is provided by AUC—Academy for Trauma Surgery (AUC—Akademie der Unfallchirurgie GmbH), a company affiliated to the German Trauma Society. The scientific leadership is provided by the Committee on Emergency Medicine, Intensive Care and Trauma Management (Sektion NIS) of the German Trauma Society. The participating hospitals submit their pseudonymized data into a central database via a web-based application. Scientific data analysis is approved according to a peer review procedure established by Sektion NIS.

The participating hospitals are primarily located in Germany (90%), but a rising number of hospitals of other countries contribute data as well (at the moment from Austria, Belgium, Finland, Luxembourg, Slovenia, Switzerland, The Netherlands, and the United Arab Emirates). Currently, approx. 30,000 cases from more than 650 hospitals are entered into the database per year. Regarding the presented study, only cases from Germany were considered.

Participation in the TraumaRegister DGU® is voluntary. For hospitals associated with TraumaNetzwerk DGU®, however, the entry of at least a basic data set is obligatory for reasons of quality assurance.

The present study is in line with the publication guidelines of the TraumaRegister DGU® and registered as TR-DGU project ID 2018–019.

### Patients

**Fig. 1:**
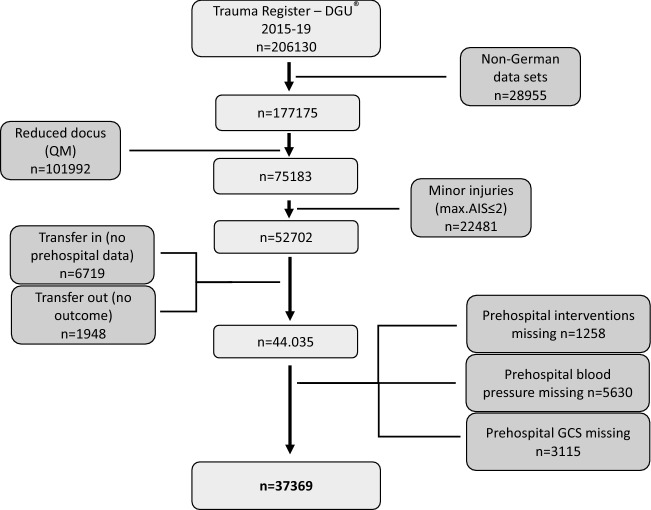
Patient inclusion

In an analysis of cases admitted to German hospitals between 2015 and 2019, patients were included if they fulfilled the following criteria (Fig. 1): major injuries (maximum Abbreviated Injury Scale ≥ 3) and complete documentation of pre-hospital interventions, blood pressure and Glasgow Coma Scale. Patients transferred in (no pre-hospital data) and those transferred out early within 48 h (no final outcome available) were excluded. Finally, a dataset of 37,369 patients remained, which was evaluated with regard to pre-hospital airway management and the underlying indications.

According to the current guidelines, the indication for an ETI was determined. We considered the following as possible indications: GCS < 9 points at scene; CPR due to cardiac arrest, shock at scene (systolic blood pressure < 90 mmHg), SpO_2_ ≤ 90% and respiratory rate (< 6/min or > 29/min).

If trauma patients presented with no other indication for ETI than shock, these cases were divided in two groups depending on whether these patients were intubated or not. Following a descriptive analysis, the standardized mortality ratio (SMR) was used as an adjusted outcome measure. The value is calculated as the quotient of observed and expected mortality rate. The expected mortality rate is based on the Revised Injury Severity Classification version II (RISC II) score [13]. This score has been developed and validated with TR-DGU data and is used for prognosis and severity adjustment in audit reports and scientific analyses.

### Statistics

Continuous data were presented as mean with standard deviation (SD) and categorical data were presented as percentages (%). Formal statistical testing in regard to the overall collective was avoided since due to the large sample size even minor differences would result in highly significant results, which could lead to over-interpretation [14]. In the subgroup with isolated shock, categorial variables were analyzed using the Chi-squared test. The Mann–Whitney *U* test was applied for comparison of continuous variables and SMR was presented with 95% confidence interval. The clinical relevance of any differences has to be carefully interpreted. When tested, a probability of less than 0.05 was considered to be statistically significant. Data were analyzed using SPSS® Statistics, version 22 (IBM Inc., Armonk, NY, USA).

## Results

### Basic demographics

As shown in Fig. 1, 37,369 patients enrolled for the present analysis, of which 11,055 (29.6%) were intubated pre-hospitally (Table 1). 70.5% of the patients were male, and the average age was 52.7 (± 22.1) years. Patients were severely injured with a mean Injury Severity Score (ISS) of 21.6 (± 11.6).

**Table 1:** Prevalence of guideline recommendations and pre-hospital ETI rate

	Overall	No pre-hospital ETI	Pre-hospital ETI
Patients, *n*	37.369	26.314	11.055
Respiratory rate			
< 6/min, *n* (%)	647 (1.7)	67 (10.4)	580 (89.4)
> 29/min, *n* (%)	631 (1.7)	343 (54.4)	288 (45.6)
SpO_2_ ≤ 90%, *n* (%)	6052 (16.2)	2809 (46.4)	3243 (53.6)
GCS at scene < 9, *n* (%)	7930 (21.2)	1118 (14.1)	6812 (85.9)
SBP < 90 mmHg at scene, *n* (%)	2173 (5.8)	890 (41)	1283 (59)
CPR, *n* (%)	1206 (3.2)	106 (8.8)	1100 (91.2)

### Indications for pre-hospital ETI

**Fig. 2:**
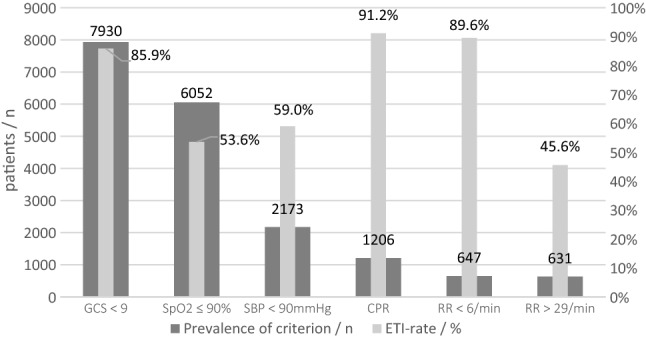
Prevalence of criterion/pre-hospital ETI rate

The recommended indications for pre-hospital airway management based on the clinically recorded parameters in comparison to the respective intubation rate are shown in Fig. 2. We see that the presence of an intubation criterion often did not necessarily lead to an ETI. 89.6% of patients with a respiratory rate < 6/min were intubated, whereas only 45.8% were intubated in case of tachypnoea > 29/min. Patients with a GCS < 9 received pre-hospital ETI in 85.9% compared to 32.4% of patients with GCS 9–13 and 9.6% of the patients with a normal GCS.

**Fig. 3:**
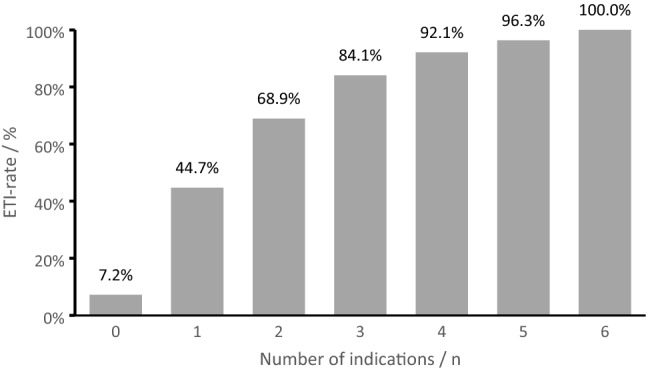
Pre-hospital ETI rate depending on number of indications

In the majority of cases (70.4%), pre-hospital ETI had not been performed. If only one criterion was present, 44% of patients were intubated. However, as the number of indications increased, the rate of pre-hospital ETI raised as well (Fig. 3).

### Endotracheal intubation in the presence of shock

Among the 1283 trauma patients who were in shock and received a pre-hospital ETI, 73.5% had multiple indications. In 582 patients, shock was the only indication, and of these, 80.4% were not intubated. The patients who got pre-hospital ETI showed a higher level of injury with a mean ISS of 26.9 (± 12.6) vs. 20.8 (± 10.8) [*p* < 0.001] and a higher rate of blunt trauma mechanisms. In case of pre-hospital ETI, the time on scene increased from an average of 25.3 min (± 12.7) to 41.6 min (± 18.0) [*p* < 0.001], whereas pre-hospital measures also increased from an average of 1.5 to 3.3.

As a consequence of hemodynamic instability, 41.2% of patients were treated with catecholamines (not intubated: 8.3%) and received a higher substitution of intravenous fluid (2314 ± 2064 vs. 1475 ± 1774), so that patients from both groups showed a stabilized circulation on admission to hospital.

Analogous to the higher level of injury patients receiving ETI showed a lower hemoglobin value (10.7 ± 2.5 vs. 12.0 ± 2.3), a higher base excess (-4.1 ± 4.7 vs. -2.7 ± 5.1) and a higher rate of coagulopathy (31.8% vs. 17.2%).

25% of patients with isolated shock were transported by Helicopter Emergency Medical Services (HEMS), increasing the pre-hospital ETI rate in this sub-cohort to 39%. Correlating with the airway management, the length of stay on ICU and standard care increased distinctively. Details are shown in Table 2.

**Table 2:** Demographic characteristics and clinical data of patients with isolated shock (SBP < 90 mmHg)

	No pre-hospital ETI	Pre-hospital ETI
Patients with isolated shock, n (%)	468 (80.4)	114 (19.6)
Age, years, mean ± SD	51.5 ± 21.4	45.9 ± 20.5
Male sex, *n* (%)	329 (70.3)	81 (71.1)
Blunt trauma, *n* (%)	384 (86.1)	94 (91.3)
Accident in traffic, *n* (%)	215 (46.0)	84 (74.3)
ISS, points; mean ± SD	20.8 ± 10.8	26.9 ± 12.6
AIS head ≥ 3 points, *n* (%)	86 (18.4)	23 (20.2)
AIS thorax ≥ 3 points, *n* (%)	206 (44.0)	66 (57.9)
AIS abdomen ≥ 3 points, *n* (%)	118 (25.2)	41 (36.0)
AIS extremities/pelvic ≥ 3 points, *n* (%)	193 (41.2)	69 (60.5)
AIS spine ≥ 3 points, *n* (%)	195 (41.7)	55 (48.2)
SBP on scene, mmHg; mean ± SD	76.6 ± 8.7	76.1 ± 8.8
SBP on admission, mmHg; mean ± SD	111.0 ± 27.4	98.5 ± 23.5
Heart rate on scene, /min; mean ± SD	90.1 ± 24.2	104.3 ± 24.4
Heart rate on admission, /min; mean ± SD	88.8 ± 22.5	99.2 ± 24.7
Use of catecholamines, *n* (%)	39 (8.3)	47 (41.2)
Substitution of intravenous fluid, ml; mean ± SD	1476 ± 1775	2314 ± 2065
Time on scene, min; mean ± SD	25.3 ± 12.7	41.6 ± 18
Rescue time, min; mean ± SD	63 ± 31	84 ± 35
Hb on admission, g/dl; mean ± SD	12 ± 2.3	10.7 ± 2.5
BE on admission, mmol/l; mean ± SD	-2.7 ± 5.1	-4.1 ± 4.7
Coagulopathy on admission, *n* (%)	77 (17.2)	34 (31.8)
Transfer with HEMS, *n* (%)	86 (18.9)	55 (50.5)
Admission to Level 1 TC, *n* (%)	353 (75.4)	103 (90.4)
Admission to Level 2 TC, *n* (%)	96 (20.5)	10 (8.8)
Admission to Level 3 TC, *n* (%)	19 (4.1)	1 (0.9)
Stay at ICU, d; mean ± SD	7.2 ± 10.3	16.5 ± 22
Stay in hospital, d; mean ± SD	19.9 ± 19.7	36.7 ± 31.6
RISC II, points; mean ± SD	7 ± 13.8	8.5 ± 15.3
Died in hospital, *n* (%)	38 (8.1)	13 (11.4)

*SD* standard deviation, *ISS* injury severity score, *ICU* intensive care unit, *SBP* systolic blood pressure, *AIS* abbreviated injury scale, *TC* trauma center, *RISC* revised injury severity classification, HEMS helicopter emergency medical service, *Hb* hemoglobin, *BE* base excess, *Coagulopathy* defined as PTT ≥ 40 s or INR ≥ 1.4, *Rescue time* defined as time from accident to admission to hospital

**Fig. 4:**
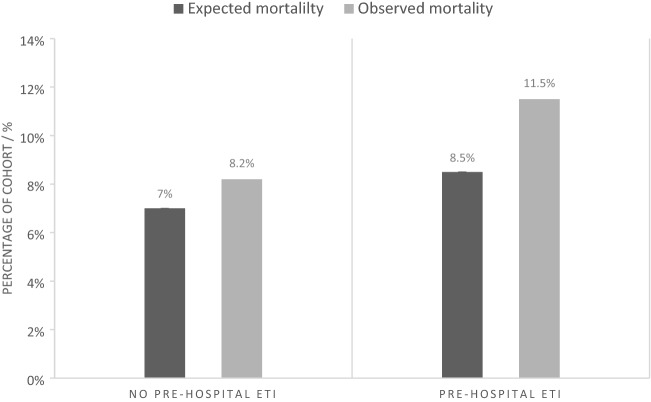
Expected mortality estimated using RISC II in contrast to observed mortality

According to RISC II, patients receiving ETI have in accordance with an increased injury severity a worse prognosis (7% ± 13.8 vs. 8.2% ± 15.3). In the presence of increased injury severity, these patients showed an increased observed mortality and with 1.35 (95% CI [0.66, 2.05]) an increased SMR compared to 1.17 (95% CI [0.81, 1.52]) [*p* = 0.6432] in non-intubated patients (Fig. 4).

## Discussion

The most important finding of this study was that ETI was not performed in the majority of trauma patients with isolated shock despite any recommendations. Pre-hospital airway management in trauma patient is of utmost importance maintaining the oxygen supply but is accompanied by certain risks and remains therefore a controversial discussed topic [15–17]. On the one hand, early intubation of critically ill and severely injured patients can improve outcome; on the other hand, attempted intubation and anesthesia, especially if not trained and performed on a regular basis, carries the risk of an increased mortality [18]. The goal of ETI in critically ill patients is a high first-pass effect [17]. Our data are from a physician-based emergency medical system, so experience in ETI has to be assumed.

In the present study, we analyzed in a first step the frequency of each guideline recommendation within the observed cohort (Table 1). While most patients with alternated respiratory rate or severe neurologic impairment received ETI according to current guidelines, the oxygen saturation or presence of shock barely influenced the intubation rate. In a second step, if patients received ETI, mainly two or more indications were present (Fig. 3). This already indicates that emergency physicians do not equally weight the guideline recommendations. Despite all recommendations and algorithms, the decision to intubate a patient contains a clinical assessment, which might not be captured in the documented data.

In a third step, patients in shock were further analyzed. In this group, 25% of the patients did not have any other indication for ETI. The comparatively low guideline coherence with an ETI rate of 19.4% might be due to an increased rate of penetrating injuries. In these patients, a “load and go” strategy might have been pursued, as a fast transport should be sought and is recommended [19]. As could be shown in contrast, the time loss due to airway management was relevant (*p* < 0.001) (Table 2).

In part, a longer time on scene can also be explained by more difficult rescue recovery, which might be suggested by higher injury severity. The TraumaRegister DGU® does not include the entrapment yet.

In addition, the concept of permissive hypotensive resuscitation has gained importance in the treatment of trauma patients without severe head injury. Patients showed improved survival due to rapid surgical bleeding control [20–23]. The current European guideline on management of major bleeding and coagulopathy following trauma recommend a restricted volume replacement strategy and a target systolic blood pressure of 80–90 mmHG [19]. Even in older patients (> 65 years), who represent a second group with high risk for traumatic injuries besides young adults and in contrast have lack of physiologic reserve, there is no evidence for synergistic effects of age and blood pressure on mortality [24, 25]. Furthermore, positive pressure ventilation can exacerbate hypotension and worsen the outcome of hypovolemic trauma patients [26]. If we now relate this to our patients, who are hypotensive but showed no relevant loss of consciousness or other indication for pre-hospital ETI, this could be an explanation for the lower mortality of the non-intubated group.

The type of transport also has an impact on the decision whether to intubate or not. Half of the intubated cohort was transferred to the providing trauma center via helicopter emergency medical service (HEMS). Here, the indication for intubation is often given more generously, as the possibilities for intervention during the flight are limited compared to ground-based transport.

Although intubation is recommended in the majority of our collective according to the guideline, 80.4% of the patients were not intubated. This is most likely explained by the clinical judgment of the physicians who decide against induction of anesthesia, intubation and possible complications in the situation. Crewdson et al. states in his retrospective study that pre-hospital intubation in awake hypotensive trauma patients has no positive effect on outcome and should be delayed until admission to the hospital. In accordance with a higher degree of injury, the ETI group showed a worse outcome in terms of length of stay on ICU and standard care as well as increased mortality compared to the non-intubated collective. A matched-pair analysis of 1200 patients from the TraumaRegister DGU® [4] showed that uncritically performed pre-hospital intubation has negative effects on the clinical course, patient care costs and the coagulation system due to higher volume substitution. Induction of anesthesia often leads to worsening hypotension and even cardiac arrest, which in turn explains the increased use of catecholamines and increased fluid substitution in our cohort [27]. However, the ETI group showed almost twice the rate of coagulopathy and decreased Hb levels on admission to hospital, whereas the acute traumatic coagulopathy is associated with a significant morbidity and mortality. To counteract this, early diagnosis, coagulation management and bleeding control is crucial, which in turn could be a reason for the higher mortality and longer stay on ICU in patients who received pre-hospital ETI. The total rescue time, which is increased according to the time on scene, can also have a negative effect on this.

On the one hand, due to differences in injury pattern, the worsened outcome as length of stay and mortality between the observed groups cannot be only justified by the intubation itself. On the other hand, an increased SMR in the ETI group indicates excess mortality in pre-hospital intubated patients.

Certain limitations of this study have to be acknowledged. This is a retrospective analysis with all the associated shortcomings, like the introduction of a selection bias, because of selective survival information and incomplete or inaccurate information. Therefore, only associations and no causalities can be derived from the underlying data. Regarding the presented study, a selection bias due to the selection of severe trauma cases has to be considered. Indication for ETI may be due to clinical decision, transport (HEMS), patient guidance/pain management, which is not reflected whether in guidelines nor in the analyzed registry data. An originally intended matched-pair study design was unfortunately not possible due to selective inclusion criteria and consecutive small number of cases describing patients with shock as remaining ETI indication. Limiting the epidemiologic validity, pre-hospital deaths are not captured in the TR-DGU. This might cause an inclusion bias, as a relevant percentage of pre-hospital deaths in trauma patients with hemorrhagic shock has to be assumed. However, almost all German hospitals participating in trauma care contribute to the register, which enables comparisons to demographic data. Summarizing, the TR-DGU enables illustration of consistency or changes in therapy or therapeutically actions.

## Conclusion

Decision making for pre-hospital intubation in trauma patients is challenging in front of a variety of factors. Despite the presence of a recommended intubation criterion, ETI is not always executed. Patients presenting with shock as remaining indication and subsequent intubation showed a decreased outcome. Whether this is only a consequence of the higher injury severity or the higher rate of complications, which could be favored by intubation and the longer rescue time, cannot be said conclusively. Further studies are needed here. Overall, isolated shock does not appear to be an appropriate indication for pre-hospital ETI, but clearly remains an important surrogate of trauma severity and the need for trauma team activation.
